# Wirtschaft und Corona: Die Bedeutung von Vertrauen in Krisenzeiten

**DOI:** 10.1007/s41358-021-00265-4

**Published:** 2021-06-21

**Authors:** Dominik H. Enste

**Affiliations:** 1 Verhaltensökonomik und Wirtschaftsethik, Institut der deutschen Wirtschaft (IW), Konrad-Adenauer-Ufer 21, 50668 Köln, Deutschland; 2 Schmalenbach Institut für Wirtschaftswissenschaften, TH Köln, Claudiusstr. 1, Köln, 50678 Deutschland; 3 IW Akademie GmbH, 10 19 42, Köln, 50459 Deutschland

## Einleitung: Unsicherheit in Zeiten von Corona

Die größte aktuelle Herausforderung ist weltweit ohne Zweifel die COVID-19 (Coronavirus SARS-CoV-2) Pandemie, die sowohl wirtschaftsethische Abwägungen (Enste und Potthoff 2020) als auch ökonomische Bewertungen (Bardt und Hüther 2021) erfordert. Angesichts der dynamischen, schwer vorhersehbaren Entwicklungen und der großen Unsicherheiten in Bezug auf die Folgen und richtigen Maßnahmen zur Eindämmung spielt jedoch für die Bewältigung der Krise das grundsätzliche Vertrauen der Menschen ebenfalls eine zentrale Rolle. Da die Fakten wie Ansteckungsrisiken, Krankheitsverläufe und Entwicklung von Impfstoffen und deren Nebenwirkungen unsicher sind, das Virus unsichtbar ist und potenzielle Gefahren durch Mutationen exponentiell steigen können, bedarf es dieses grundsätzlichen Vertrauens in die Institutionen und handelnden Personen, gerade wenn weitreichende Eingriffe in die persönliche Freiheit und wirtschaftlichen Prozesse bisher ungekannten Ausmaßes stattfinden. Das Vertrauen in das Gesundheitssystem ist dabei das eine, das Vertrauen in die langfristige, nachvollziehbare und transparente Kommunikation der Politik, den Zusammenhalt der Gesellschaft und die Robustheit und Leistungsfähigkeit der Wirtschaft – zum Beispiel bei der Impfstoffentwicklung und der Sicherheit der Grundversorgung – das andere.

## Makroebene: Vertrauen im internationalen Ländervergleich und in Deutschland

Unser internationaler Ländervergleich zeigt, welche Länder hier besser für die Krise gerüstet waren als andere (Enste und Suling, 2020). Dabei geht es weniger um die ökonomischen Maßnahmen und die finanziellen Möglichkeiten der Länder und Regierungen als vielmehr um das fragile, psychologische Konstrukt des Vertrauens. Denn Vertrauen sorgt nicht nur in guten Zeiten für mehr Wohlstand und Wachstum, sondern in schlechten Zeiten für die notwendige Verlässlichkeit, um Panik zu vermeiden. Allerdings lässt sich Vertrauen nur schrittweise und langfristig aufbauen, dafür aber umso schneller verspielen (Algan et al. 2017, S. 317; Newton und Zmerli, 2011). Außerdem zahlt sich Vertrauen in Mitmenschen und Institutionen nicht generell aus, sondern nur in Ländern, die insgesamt über ein hohes Sozialkapital (Putnam 1993) verfügen. In solchen Ländern profitieren Menschen, die ihren Mitmenschen grundsätzlich vertrauen und erzielen höhere Lebenseinkommen und sind insgesamt zufriedener mit ihrem Leben (Stavrova und Ehlebracht 2016).

Enste und Suling (2020) untersuchten im IW-Vertrauensindex 20 Länder im Hinblick auf das Vertrauen in Wirtschaft, Staat und Gesellschaft über den Zeitraum von 2000 bis 2018 – also vor der Corona-Krise – anhand von 15 Indikatoren wie dem Ausmaß des Vertrauens gegenüber Mitbürgern, der Regierung, verschiedenen Wirtschaftsbranchen und in die Wirtschaft generell. Bei allen statistischen Schwierigkeiten, die ein solcher Index mit sich bringt, zeigt er – über die zufälligen und nicht erfassten Faktoren hinaus – warum die Krise in den Ländern unterschiedlich behandelt und wahrgenommen wird. In den skandinavischen Ländern sowie den Niederlanden, Deutschland und der Schweiz herrschte vor der Krise ein ausgeprägtes Klima des Vertrauens. Das politische System, das Wirtschaftssystem und die Gesellschaft wurden – im Großen und Ganzen – als zuverlässig und vertrauenswürdig wahrgenommen. Auf Platz 1 des Vertrauensindex lag Dänemark mit 91 von 100 möglichen Punkten. Deutschland lag mit 74 Punkten auf Platz 7 – hinter Finnland (89), Schweden (86), den Niederlanden (82), der Schweiz (80) und Irland (76). Die südeuropäischen Staaten Griechenland (8), Italien (30), Spanien (38) und Portugal (43) landeten am Ende des Rankings. Das Misstrauen gegenüber der Politik und der Regierung waren hier besonders ausgeprägt. So vertrauen die Menschen in Griechenland (0) und Italien (10) dem politischen System de facto gar nicht. Vertraut wird nur im engen Verwandten- und Freundeskreis. Generalisiertes Vertrauen in zum Beispiel die Unbestechlichkeit von Institutionen wird hier eher bestraft. Das erschwerte die Zusammenarbeit bei der landesweiten oder internationalen Krisenbewältigung wie der Corona-Pandemie erheblich.

Eine aktuelle Studie der Bertelsmann Stiftung (Brand et al. 2021) beschreibt, wie sich das Vertrauen und der gesellschaftliche Zusammenhalt während der Krise in Deutschland entwickelt hat. Sie zeigt, dass im Jahresverlauf 2020 der gesellschaftliche Zusammenhalt bis zur Jahresmitte stabil geblieben ist. Die Wahrnehmung des gesellschaftlichen Miteinanders wurde sogar positiver als zu Jahresbeginn eingeschätzt. Dieser Trend kehrt sich im zweiten Halbjahr um und sank mit dem zweiten Lockdown zum Jahresende 2020 noch unter das Niveau zu Jahresbeginn ab. Insbesondere Menschen in prekären Lebensverhältnissen sind skeptischer geworden und fürchten um den Zusammenhalt und das Sozialkapital. Mehr als die Hälfte der Menschen mit niedrigem sozioökonomischen Status macht sich zudem große Sorgen um die eigene Zukunft. Aber auch in der Mittelschicht sind über 40 % – und damit doppelt so viele wie noch im Mai/Juni 2020 – in großer Sorge um ihre Zukunft. Außerdem fühlen sich vor allem die jungen Menschen unter 30 Jahren um ihre Zukunft betrogen (66 %) und 71 % fühlen sich einsam und verloren. Jenseits dieser Entwicklungen bleibt aber das generelle Vertrauen in die Mitmenschen recht stabil. Dies könnte somit eine gute Basis für mehr Vertrauen auch von Seiten der Politik in die Bürgerinnen und Bürger sein.

## Strategische Entscheidung: Vertrauen oder Kontrolle

Das zentrale Ziel bei der Corona-Pandemie ist, die Verbreitung des Virus zu stoppen. Dafür ist es notwendig, dass – neben der Impfung und der Entwicklung von Medikamenten gegen die Symptome – die Menschen weniger engen Kontakt haben. Notwendig ist deshalb eine Verhaltensänderung der Menschen, die in vielen Lebensbereichen stattfinden muss. Dazu gehört nicht nur der öffentliche Raum, sondern insbesondere auch der private Raum, Büros, Industriegelände und der gesamte Kulturbetrieb sowie Restaurants, Hotels und Freizeitanlagen. Änderungen im Verhalten lassen sich grundsätzlich auf zwei Wegen erreichen: durch Zwang und Anreize oder durch Einsicht und freiwilliges Befolgen der Regeln. Mischformen eignen sich dabei nicht, wie zum Beispiel die Erforschung der Steuermoral und die Bereitschaft, Steuern zu zahlen, im Rahmen des Slippery Slope Modells zeigt (Kirchler und Muehlbacher 2010). Steuerzahlungen lassen sich entweder mit Macht und erzwungener Kooperation eintreiben oder mit Vertrauen und freiwilliger Kooperation. Ganz ähnliches gilt auch für die Pandemiebekämpfung, bei der ja auch ein Beitrag zum Kollektivgut mit nur geringem eigenen Nutzen gefordert ist – insbesondere von jüngeren Menschen unter 30 Jahren, die extrem geringeres Sterberisiko durch Corona aufweisen. Von den rund 86.000 Menschen, die an oder mit Corona verstorben sind, waren nur 86 Menschen, die unter 30 Jahren alt waren (Robert Koch-Institut 2021).

Vertrauen oder Kontrolle als Basis für Verhaltensänderungen gilt für Staaten/Regierungen im Umgang mit Bürgerinnen und Bürgern, für Unternehmen und Organisationen im Umgang mit ihren Mitarbeiterinnen und Mitarbeitern (Enste und Suling 2020) aber letztlich auch im privaten Miteinander oder bei der Erziehung der Kinder (ausführlicher Enste et al. 2020). Die Bundesregierung hat sich hier recht frühzeitig für eine Misstrauenskultur entschieden und den Fokus auf die Belehrung, Kontrolle und zwangsweise Einschränkung der Kontakte mithilfe von Verordnungen, Gesetzen und zuletzt einer bundeseinheitlichen Regelung in Form der Verschärfung des Infektionsschutzgesetzes gelegt. Aufgrund des „Vorbilds“ China und der Unsicherheiten im März 2020 war dies anfangs nachvollziehbar; aber im Zuge der weiteren Entwicklung wäre im Sommer 2020 ein Schwenk in Richtung mehr Vertrauen und Selbstverantwortung statt mehr Ordnungsrecht und pauschalen Lockdowns möglich und sinnvoll gewesen. Aber der Fokus lag darauf, jedes Risiko zu vermeiden.

Außerdem neigen Menschen dazu, das Fehlverhalten einzelner deutlich eher wahrzunehmen als das korrekte Verhalten vieler Menschen. Wenn sich 90 % (freiwillig) an Regeln halten, weil sie die Sinnhaftigkeit einsehen, werden diese dennoch mit bestraft, wenn wegen der fehlenden 10 % generelle Verbote (wie Ausgangssperren) erlassen werden, deren Wirksamkeit zudem nie valide empirisch nachgewiesen wurden. Dabei wäre es notwendig hier differenziert diejenigen zu bestrafen, die sich nicht an die allgemeinen Regeln der Kontaktbeschränkungen oder die Maskenpflicht in engen Räumlichkeiten halten. Ein Mikromanagement und differenzierte Maßnahmen ab bestimmten (vielfach als willkürlich wahrgenommenen) Inzidenzwerten setzt das auf Misstrauen basierende Management fort. Dies führt nachweislich zu schlechteren Ergebnissen – zumindest gemäß den Forschungsergebnissen auf Unternehmensebene (Enste et al. 2020), in experimentellen Designs (ausführlicher Enste et al. 2020) und auch auf individueller Ebene (Fetchenhauer und Dunning 2009).

Auf staatlicher Ebene wurden natürliche Experimente während der aktuellen Pandemie zur Überprüfung der Wirksamkeit von Maßnahmen und Regimen leider nicht durchgeführt. Ein Kontrollregime bedingt hohe Transaktionskosten und hat mit dem Problem zu kämpfen, dass in freiheitlichen und demokratischen Ländern eine vollständige Kontrolle unmöglich ist. Allenfalls in Diktaturen oder Ländern mit starker staatlicher Macht und Kontrolle wie in China, Singapur oder Südkorea lassen sich mit flächendeckender Überwachung diese Zwangsregime zur Pandemiebekämpfung umsetzen. In liberalen Gesellschaften funktioniert eine vertrauens- und einsichtsbasierte Bekämpfung sehr viel besser und ist mit Blick auf die Grundrechte und andere wichtige gesellschaftliche Ziele neben dem Gesundheitsschutz auch geboten.

## Analyse: Warum Vertrauen so wichtig ist

Laotse soll schon vor über 2500 Jahren gesagt haben: „Wer nicht vertraut, dem vertraut man nicht.“ Damit andere Vertrauen schenken, ist zunächst die eigene Vertrauenswürdigkeit gefragt. Vertrauen aufzubauen, ist dabei ein langwieriger Prozess. Leider ist es nur allzu menschlich, sich vor allem daran zu erinnern, dass jemand Vertrauen missbraucht hat. Dadurch entsteht der Eindruck, andere Menschen seien weniger vertrauenswürdig als dies eigentlich der Fall ist. Auch für Medien, Öffentlichkeit und Leser sind Nachrichten über Fehlverhalten, Misstrauen und Vertrauensbruch interessanter als Berichte über Erfolge im Kampf gegen Corona. Dies verstärkt den Effekt zusätzlich, dass die Vertrauenswürdigkeit anderer Menschen unterschätzt wird. Gerade in Krisenzeiten ist aber dieses Vertrauen gefragt, auch wenn Deutschland hier vor der Krise vergleichsweise gut aufgestellt war.

Menschen müssen Vertrauen in die Notwendigkeit von Freiheitseinschränkung und die Beachtung zum Beispiel von Quarantäne-Maßnahmen, auch ohne Krankheitssymptome, haben. Denn häusliche Quarantäne lässt sich in Deutschland nur mit hohem Aufwand kontrollieren. Wenn aber die Entdeckungswahrscheinlichkeit gering ist, sind auch Freiheitsstrafen bis zu 5 Jahren oder hohe Geldstrafen kaum abschreckend. Entscheidend ist das generalisierte Vertrauen in die Mitmenschen, dass sie sich solidarisch verhalten, eine persönliche, drastische Einschränkung hinnehmen, um der Gesellschaft bei der Eindämmung der Epidemie als Kollektivgutproblem zu helfen. Private Zusammenkünfte lassen sich ebenfalls kaum kontrollieren und mit Ausgangssperren nur bedingt vermeiden, wenn diese dann vor 22 Uhr beginnen und erst um 5 Uhr am nächsten Tag enden, wie dies u. a. Studierendengruppen aus ihren Erfahrungen zum Beispiel in den Niederlanden, Frankreich oder Spanien berichteten.

Außerdem ist Vertrauen in die Fakten und Maßnahmen von Autoritäten erforderlich. Dabei ist es problematisch, wenn das Robert Koch-Institut aber auch Universitäten und Forschungseinrichtungen suggerieren, sie wüssten, was richtig ist und es gäbe „die“ Wissenschaft. Eine glaubwürdige Strategie und Kommunikation, die auch Unsicherheiten zugibt angesichts der dynamischen Entwicklungen, ist in Ländern ohne umfassende Kontrollmöglichkeiten langfristig erfolgreicher (Enste und Suling 2020). Eine langfristige Strategie, evidenzbasierte Argumente und Zuversicht in Kombination mit der Übernahme von Verantwortung wirken sowohl im Unternehmenskontext besser als auch auf staatlicher Ebene. Verantwortung und Vertrauen hängen eng zusammen. Eine Politik des „C.Y.A.“ („Cover your ass“), bei der jeder nur darauf bedacht ist, letztlich nicht rechtlich verantwortlich gemacht werden zu können, sorgt insbesondere in der öffentlichen Verwaltung und großen Konzernen für ineffiziente, ineffektive, defensive Entscheidungen und zu einer Fehlervermeidung, die innovative Lösungen ausschließt (ausführlicher Artinger et al. 2019).

## Fazit: Vertrauen in Unternehmen und Marktwirtschaft

Die gewählte Strategie der Pandemiebekämpfung mithilfe des Ordnungsrechts und basierend auf Kontrollen, fußt auf der Annahme, dass Menschen nur durch Zwang und Bestrafung oder Anreize zu Verhaltensänderungen zu bewegen sind. Diese zum einem vom Menschenbild des Homo oeconomicus und zum anderen stark juristisch geprägte Sichtweise führte zu ausgefeilten, seitenlangen Gesetzestexten und konkretisierenden Verordnungen und hohen Strafandrohungen, die in vielen Fällen nicht logisch und nicht nachvollziehbar waren und zum Beispiel zu Ungleichbehandlungen innerhalb der Wirtschaft geführt haben. Dies betrifft die Regelungen hinsichtlich des Lockdowns und der Schließung von Geschäften, aber auch die Regelungen zur Kompensation der Umsatzausfälle. Wenngleich viele Milliarden an Steuergeldern zur Sicherung von Arbeitsplätzen und zum Überleben von Unternehmen ausgegeben wurden, gibt es viele Verlierer, deren Geschäftsmodelle nach Monaten des Lockdowns nicht mehr funktionieren.

Damit soll nicht in Frage gestellt werden, dass der Schutz von Menschenleben und die Eindämmung der Verbreitung des Virus notwendig und unabdingbar gewesen sind. Was fehlte – insbesondere vor dem zweiten und dritten Lockdown – war eine breitere interdisziplinäre Fundierung der Maßnahmen und die Suche nach alternativen Wegen, sowie eine empirische Evidenz für die Wirksamkeit der staatlichen Zwangsmaßnahmen. Dabei hätte die Politik und öffentliche Verwaltung viel von der Wirtschaft lernen können. Unternehmen haben angesichts der Megatrends wie Demographischer Wandel, Individualisierung und Digitalisierung frühzeitig erkannt, dass die Mitarbeiterführung mit einem neoklassischen Menschenbild und einer Misstrauenskultur langfristig für viele Mitarbeiter nicht funktioniert (Enste et al. 2020). Die Analysen verdeutlichen, dass eine Verantwortungs- und Fehlerkultur die Vertrauenskultur stärkt und dazu führt, dass Menschen freiwillig kooperieren und im Sinne der Gemeinschaft agieren, auch ohne regelmäßige Kontrollen. Damit ist kein naives, blindes Vertrauen gemeint, sondern eine wertschätzende Kontrolle für eigenverantwortlich tätige Mitarbeiter.

Übertragen auf die staatliche Ebene würde ein allgemeiner Rahmen gefordert mit nachvollziehbaren, logischen Regeln und dem grundsätzlichen Vertrauen in mündige Bürger und deren Selbstverantwortung. Wie in Unternehmen auch, müssen Fehlverhalten und Verstöße gegen diese allgemeinen Regelungen sanktioniert werden. Der Vorteil dieses Weges bedeutet weniger Kosten durch pauschale Lockdowns, weniger generelle Freiheitseinschränkungen und zugleich eine größere Effektivität der Bekämpfung, zumindest wenn sich die Ergebnisse aus den Unternehmen (Enste et al. 2020) und aus der Forschung rund um Steuerhinterziehung (Kirchler und Muehlbacher 2010) hier übertragen lassen. Leider gibt es bisher auf staatlicher Ebene keine verlässlichen empirischen Befunde, welche Pandemiebekämpfungsstrategie langfristig erfolgreicher ist. Aber die Schweiz, Schweden und die Niederlande haben mit ihrer Strategie, mehr auf Selbstverantwortung zu setzen, ähnliche Entwicklungen bei den Fallzahlen und Sterberisiken erzielt wie Deutschland (Ourworldindata.org 2021).

Angesichts des von vielen beklagten Staatsversagens wird deutlich, wie erfolgreich marktwirtschaftlich organisierte Prozesse bei der Lösung von Knappheiten sind. Staatsversagen zeigt sich bei den „Jo-Jo-Effekten der Lockdowns und Lockerungen sowie dem kompletten Wirrwarr, wer nun wann was darf oder nicht.“ (Straubhaar 2021). Dies sei der „Offenbarungseid, dass die Staatswirtschaft nicht halten kann, was sie verspricht.“ (Straubhaar 2021). Ursachen dafür sind aus Sicht der Ökonomik falsche Anreize in der Verwaltung, fehlender Wettbewerb, Eigenleben von Verwaltung und Politik sowie ausufernde Bürokratie, die u. a. erklären, warum eine Staats- und Planwirtschaft scheitern muss. Die Pandemie hat dies leider bestätigt.

Die Soziale Marktwirtschaft hat hingegen funktioniert. Bei der Absicherung durch Build-In-Stabilisatoren wie dem Kurzarbeitergeld konnten insbesondere niedrige Einkommensschichten vor Einkommenseinbußen erfolgreich geschützt werden, so dass die Ungleichheit sogar gesunken ist (ausführlicher Beznoska et al. 2020; Grabka 2021). Zugleich hat die (Markt‑) Wirtschaft auch in Krisenzeiten ihre Resilienz bewiesen und unter schwierigen Bedingungen zuverlässig die Versorgung der Bevölkerung mit Nahrungsmitteln, Strom, Internet, Homeoffice-Equipment usw. sichergestellt. Nicht zuletzt hat der Kapitalmarkt genügend Risikokapital bereitgestellt für die Impfstoffentwicklung. Neben den wenigen erfolgreichen Unternehmen wie Biontech SE, die nun die ersten Impfstoffe liefern, gibt es viele Verlierer, bei denen Investoren ihr Risikokapital verloren haben. Aber dieser Wettbewerb hat sichergestellt, dass es nun in Kürze ausreichend Impfstoffe geben wird. Sofern von Beginn an mehr Impfstoff bestellt worden wäre – auch um ihn dann an Länder wie Indien weiterzugeben, die über weniger finanzielle Ressourcen verfügen, wären frühzeitiger noch mehr Kapazitäten aufgebaut worden. Vielleicht hätten im Projektmanagement erfahrene Unternehmen und in der Logistikbranche erfolgreiche Manager auch an anderen Stellen das staatliche Pandemiemanagement effizienter und effektiver gestalten können. Generell veranschaulicht die Krise, dass mehr Vertrauen in (Markt‑) Wirtschaft unter klaren ordnungspolitischen Rahmenbedingungen und mehr Vertrauen in die Menschen (fast) überall eine gute Idee ist.
